# The importance of selecting the correct site to apply spinal manipulation when treating spinal pain: Myth or reality? A systematic review

**DOI:** 10.1038/s41598-021-02882-z

**Published:** 2021-12-03

**Authors:** Casper G. Nim, Aron Downie, Søren O’Neill, Gregory N. Kawchuk, Stephen M. Perle, Charlotte Leboeuf-Yde

**Affiliations:** 1 Medical Research Unit, Spine Center of Southern Denmark, University Hospital of Southern Denmark, Odense, Denmark; 2 Department of Regional Health Research, University of Southern Denmark, Odense, Denmark; 3 Department of Chiropractic, Faculty of Medicine, Health and Human Sciences, Macquarie University, Macquarie Park, Australia; 4 Department of Physical Therapy, Faculty of Rehabilitation Medicine, University of Alberta, Edmonton, Canada; 5 School of Chiropractic, College of Health Sciences, University of Bridgeport, Bridgeport, USA

**Keywords:** Musculoskeletal system, Musculoskeletal system, Rehabilitation

## Abstract

The concept that spinal manipulation therapy (SMT) outcomes are optimized when the treatment is aimed at a clinically relevant joint is commonly assumed and central to teaching and clinical use (candidate sites). This systematic review investigated whether clinical effects are superior when this is the case compared to SMT applied elsewhere (non-candidate sites). Eligible study designs were randomized controlled trials that investigated the effect of spinal manipulation applied to candidate versus non-candidate sites for spinal pain. We obtained data from four different databases. Risk of bias was assessed using an adjusted Cochrane risk of bias tool, adding four items for study quality. We extracted between-group differences for any reported outcome or, when not reported, calculated effect sizes from the within-group changes. We compared outcomes for SMT applied at a ‘relevant’ site to SMT applied elsewhere. We prioritized methodologically robust studies when interpreting results. Ten studies, all of acceptable quality, were included that reported 33 between-group differences—five compared treatments within the same spinal region and five at different spinal regions. None of the nine studies with low or moderate risk of bias reported statistically significant between-group differences for any outcome. The tenth study reported a small effect on pain (1.2/10, 95%CI − 1.9 to − 0.5) but had a high risk of bias. None of the nine articles of low or moderate risk of bias and acceptable quality reported that “clinically-relevant” SMT has a superior outcome on any outcome compared to “not clinically-relevant” SMT. This finding contrasts with ideas held in educational programs and clinical practice that emphasize the importance of joint-specific application of SMT.

## Introduction

Clinical guidelines recommend spinal manipulative therapy (SMT) as one possible intervention for spinal pain but do not provide specific details about how or where to deliver the intervention^1,2^. These generic recommendations do not consider the potential importance of applying manipulation at a specific application site, albeit such factors are considered important by many clinicians using manual therapy^3^. Much attention is invested in learning to determine the appropriate vertebral level, side, and thrust style (force–time profile), as this is believed to be important for clinical outcomes. SMT is, therefore, considered a highly skilled procedure that can only be mastered with extensive training. Consequently, the concept of treatment specificity of SMT is emphasized in clinical education^3,4^.

But what is, in fact, the evidence for this approach in relation to clinicians being able to identify a clinically relevant vertebra and that SMT produces a specific effect on or around this joint? A major hindrance to finding the exact site to treat is the poor diagnostic performance of many clinical tests used to locate aberrant spinal function^5,6^. On the other hand, once a segment has been selected, laboratory-based (animal) research has found biomechanical effects on the tissues and cell structures specific to the application site of SMT. For example, spinal stiffness at the treated vertebral level decreased at a higher rate at the site of SMT as compared to an adjacent vertebral level^7^. Similarly, higher muscle spindle discharge has been reported at the treated vertebral level than at the adjacent level^8^. However, it is unclear if such findings translate to humans and whether they have any clinical relevance. If indeed such effects are joint-specific in a clinical (human) context and of clinical relevance, the clinical outcomes would arguably differ depending on the SMT application site.

Therefore, the purpose of this review of clinical studies was to compare spine-related outcomes when SMT was applied at a candidate site presumed to be clinically relevant vs. when SMT was applied at any other spinal location.

### Objectives

We explored whether SMT applied at a candidate site is superior to SMT applied at a non-candidate site in relation to the clinical outcome. Our primary outcome was between-group differences in patient-reported outcomes (e.g., pain intensity or disability). Secondary outcomes included objective measurements (e.g., pressure pain detection threshold (PPT) and range of motion).

## Materials and methods

### Design

This systematic review was submitted to *The international prospective register of systematic reviews* (PROSPERO) (ID = 202598). Minor additions were made to the protocol after registration. These included clarification of definitions for “candidate site” and “non-candidate site” and the addition of four items to rate study quality (in addition to the existing Cochrane risk of bias tool). The manuscript was prepared according to the *Preferred Reporting Items for Systematic reviews and Meta-Analyses* (PRISMA2020) statement^9^. We have not published the protocol of the systematic review.

### Eligibility criteria

SMT was defined as a high velocity, low amplitude force. This can be applied using two methods, either manually or via some instrument (e.g., an impulse device or a robotic arm). We included only randomized controlled study designs on humans with spinal pain in any region and of any duration, comparing SMT applied to any candidate site compared to SMT applied to any non-candidate site, where the between-group effect sizes were reported or estimable.

Non-thrust mobilization techniques (e.g., Maitland grades I through IV)^10^ were excluded. We also excluded studies that used different SMT applications (i.e., studies that compared manual SMT with any instrument-induced SMT) and studies in which some additional treatment was given to only one group. We also excluded studies that compared any SMT to sham SMT. Eligible studies had to be published in English or possible to be translated to English by a research team member. However, we did not find any relevant non-English articles.

The application site was determined as to where the treating clinician attempted to apply the force thrust of the SMT. We defined the candidate site as the SMT site determined to be relevant for clinical outcomes as i) prescribed by the treating clinician, regardless of the method used, or ii) if the clinician had to follow a procedure defined in a study protocol regardless of the method prescribed. As described above, the non-candidate site was SMT applied elsewhere in the spine but with no clinical indication.

We compared candidate SMT sites to the following three types of non-candidate SMT sites:

(i) SMT at the candidate site compared to SMT to the opposite side of the indication (i.e., at the same spinal level but on the contralateral side—“same level”)
(ii) SMT at the candidate site compared to SMT elsewhere in the same spinal region (i.e., cervical, thoracic, or lumbar—“same region”)
(iii) SMT at the candidate site compared to SMT to a distant spinal region (“remote region”)

### Search for literature

We systematically searched the literature in four electronic databases: PubMed, Embase, Index to Chiropractic Literature, and CINAHL from earliest to September 15th, 2020. The search strategy was initially developed for PubMed (S1) and afterward adopted to other databases in collaboration with a research librarian from the University of Southern Denmark. The search contained terms relating to (i) spinal pain, (ii) SMT applied at candidate sites, and iii) non-candidate SMT sites. MeSH terms and truncation (*) were elected as appropriate, allowing us to search multiple terms and portions of similar words.

### Study selection

We used Covidence^11^ to handle the screening of potentially relevant studies. Titles and abstracts for all identified studies were screened for inclusion independently by two authors (CGN and AD), with differences discussed until consensus was reached. If consensus could not be reached, a third author would arbitrate the decision (CLY). After screening, the same two authors reviewed the relevant full texts until consensus was reached. If consensus could not be reached, the same third author would arbitrate the decision. However, no third opinions were necessary. Finally, CGN manually applied backward citation chaining by reviewing the references of each included study to identify potential additional studies.

### Data extraction

One author (CGN) extracted data from included studies. A second author (SON) verified data extraction, resolving any discrepancy through consensus with a third author (AD). Data extraction included: study description, participant characteristics, description of intervention and control therapies, and outcome measurements at all time points. We extracted the between-group differences for all outcomes reported at all time points. If between-group differences were not reported, we calculated Cohen’s effect sizes based on the reported mean within-group changes in the SMT arms ([mean_candidate_ − mean_non-candidate_]/SD_pooled_)^12^. We extracted only patient-reported outcomes if we had to calculate the effect sizes from the within-group changes due to statistical uncertainty about the assumptions^12,13^. Finally, if a study presented multiple different outcomes for the same domain (e.g., PPT at multiple regions), we extracted only the first reported result (e.g., PPT at the right arm).

We defined patient-reported outcomes as a subjective measurement if reported by the patient^14^ and objective measurements as assessments that are not subject to a large degree of subjective interpretation^15^. If > 20% of the data were missing, we did not extract that outcome. If it was apparent that outcome data necessary to compute between-group differences had been collected without being reported, we contacted the lead author to request the data.

### Risk of bias and quality assessment

Each study was assessed for risk of bias by two authors independently (CGN (100%) and AD (50%), or CLY (50%)) using the Cochrane Risk of Bias tool (RoB) 1^16^. We modified item (iii) “blinding of participants and personnel,” given that both study arms received SMT. Instead, we assessed whether the participants were naïve to SMT. Item (vii) “other sources of bias” assessed if the statistical analysis was performed in a blinded manner. The items are listed below with a description for “low risk of bias”:

(i) Random sequence generation (i.e., reported that there was some independent sequence generation (including coin toss))
(ii) Allocation concealment (i.e., reported that the allocation to study group was concealed to the assessor/clinician)
(iii) Participants were naïve to SMT (i.e., the study subjects should be new to SMT or have no interest in the outcome. If they were likely to have been previous patients, the treatment must be such that they were unlikely to discern the difference between the candidate and the non-candidate site, thus considered to be effectively ‘blinded’ and unlikely to somehow ‘guide’ the outcomes)
(iv) Blinding of outcome assessment (i.e., blinding of outcome assessors)
(v) Incomplete outcome data (i.e., the drop-out rate must be clearly reported or discernible within the tables of results and not exceeding 20%)
(vi) Selective reporting (i.e., all planned outcome variables reported in the Methods section must be reported in the Result section, and if available, also to be consistent with any trial registration or published protocols)
(vii) Other sources of bias (this included blinded statistical analysis)

Authors (CGN, AD, CLY) undertook to pilot the risk of bias tool before independent assessment. Each item was reported as having “low” or “high” risk of bias and was considered to have “high risk” if the item was not reported. If we were unsure of an item, the item was reported as “unsure”. If consensus could not be reached, a third author (SON) would arbitrate the decision.

#### Risk of bias per study

The individual study’s overall RoB was considered to be “low risk” if there was a maximum of one “high risk” item and one “unsure” item. “Moderate risk” was defined if there were a maximum of two “high risk” items and one “unsure” item, and all other combinations were considered as “high risk”. This judgment was visualized using colors “low risk” (green), “unsure” (yellow), and “high risk” (red).

#### Risk of bias per item

We also collated the RoB for all included studies at the level of each item, using the same color labeling system. An item was considered to have a “low risk” of bias if it had a maximum of 2 red/yellow included studies, “moderate risk” if it had a maximum of 3 red/yellow included studies, and “high risk” for all the others.

The RoB is presented visually, and the figures were created in R vers. 4.1^17^ for Ubuntu 20.04, using the add-on package *dmetar*^18^.

#### Quality assessment

In addition to the RoB tool, the following items were used to assess individual study quality^19,20^.

The quality assessment items were added, given that risk of bias assessment (alone) would not sufficiently capture study quality.

(i) The SMT was sufficiently well described to be reproducible
(ii) The experience of the investigator/therapist was sufficient to ensure competence in the delivery of SMT (e.g., not delivered by students)
(iii) The primary outcome of the study was stated to have been validated. We considered pain and disability to be valid, regardless of whether this was stated in the article, as both are considered core outcomes in spine pain research^21^.
(iv) The statistical analysis was reported to a sufficient level to facilitate re-analysis

Each item was marked as ‘yes’, ‘unsure’, or ‘no’. To be considered acceptable quality overall, studies had to satisfy ‘yes’ for at least items (iii) and (iv).

#### Study credibility

An individual study was considered credible if assessed as having either low or moderate RoB and acceptable quality.

### Data synthesis

The synthesis is reported according to the *Synthesis Without Meta-analysis (SWiM)* in Systematic Reporting Guideline^22^. It was not possible to pool the results for meta-analysis due to heterogeneity in study design, the SMT application, and participant characteristics. We intended to report the differences in outcomes for the three control groups (“same level”, “same region”, or “remote region”) by counting the statistically significant between-group differences for all estimates. When interpreting results, we prioritized credible studies (low/moderate RoB and acceptable quality). All results are reported in tables.

## Results

### Description of studies

**Figure 1:**
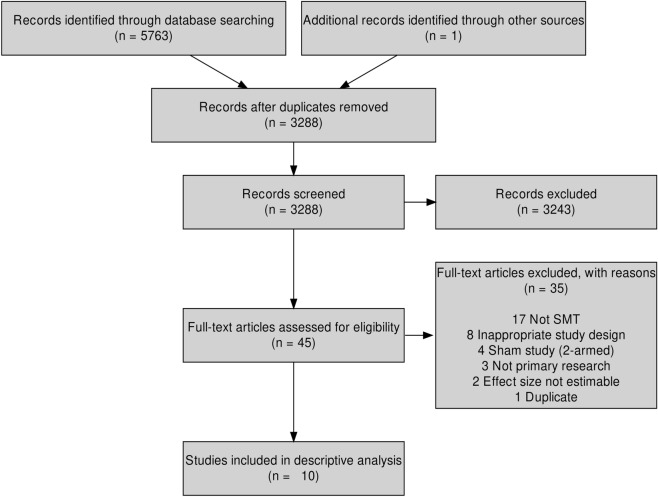
PRISMA flow diagram of the literature search and study inclusion in a systematic review comparing the outcome of applying spinal manipulative therapy at a candidate site versus a non-candidate site.

As shown in Fig. 1, we screened 3,288 articles, from which nine were included for analysis^23–31^. One additional article was found using backward citation tracking^32^, which resulted in ten included studies. All articles were in English and published between 2003 and 2020. All but three authors^25–27^ reported if there were any conflicts of interest, and four reported that they received funding^23,24,31,32^. We contacted the authors of three articles^30,33,34^ with insufficient data to estimate effect sizes. We received one response that allowed us to include that article^30^.

Table 1 lists descriptive information for each study. The study population ranged from 39 to 186, including patients with either cervical pain (n = 6) or lumbar pain (n = 4). Five studies included chronic pain patients, two included acute pain patients, and three did not specify this. The number of SMT sessions ranged from 1 to 10, and, most often, the outcomes were assessed immediately thereafter (n = 6). All but one study included patient-reported outcomes. Seven studies reported between-group differences for objective outcomes, most commonly PPT (n = 3). Four studies did not report between-group estimates. Therefore, we calculated effect sizes from the reported within-group differences^25,26,30,32^. No outcomes were excluded due to having more than 20% missing data.

**Table 1:** Description of 10 studies included in a systematic review comparing the outcome of applying spinal manipulative therapy at a candidate site versus a non-candidate site.

References	- Country - Setting	- Participants - Recruitment	Total number of participants (Candidate site/non-candidate site)	Candidate site vs non-candidate site - Same level - Same region - Remote region	Outcomes promised in method - Same outcomes reported in the results	Number of SMT sessions	Add-on intervention for both groups
Haas et al.^23^	- USA - Chiropractic college outpatient clinic	- Adult neck pain patients (duration not specified) - Referral or advertisement	99 (47/52)	Cervical SMT vs. Random computer generated application - Same region	Subjective: Cervical pain intensity and stiffness - Yes	1	–
Cleland et al.^24^	- USA - Military Health System, and out-patient physical therapy	- Adult acute low back pain patients who fit an SMT clinical prediction rule - Military Health System and out-patient practice	75 (38/37)	Lumbar SMT vs. Non-specific application - Same region	Subjective: Lumbar pain intensity and disability - Yes	2	Daily: Range of motion exercise program and stretching
Sutlive et al.^25^	- USA - Military hospital	- Adult acute low back pain patients who fit an SMT clinical prediction rule - Military hospital	60 (30/30)	Lumbar SMT vs. Non-specific application - Same region	Subjective: Lumbar pain intensity and disability - Yes	1	Twice a day for 30 s: A pelvic tilt range of motion exercise
Martinéz-Segura et al.^26^	- Spain - Private physiotherapy practice	- Adult bilateral chronic mechanical neck pain patients - Private practice	62 (29/33)	Cervical SMT vs. Thoracic SMT - Remote region	Subjective: Cervical pain intensity Objective: Cervical range of motion and pressure pain threshold - Yes	1	-
de Oliveira et al.^27^	- Brazil - Private physiotherapy practice	- Adult non-specific chronic low back pain patients - Private practice	148 (74/74)	Lumbar SMT vs. Thoracic SMT - Remote region	Subjective: Lumbar pain intensity Objective: Lumbar Pressure pain threshold - Yes	1	-
Karas and Olson Hunt^28^	- USA - Hospital orthopedic department	- Adult with neck pain (duration not specified) - Out-patient hospital	39 (19/20)	Thoracic SMT vs. Non-specific SMT - Same region	Subjective: Cervical pain intensity Objective: Cervical range of motion - Yes	1	-
Bautista-Aguirre et al.^29^	- Spain - Private physiotherapy practice	- Adult chronic mechanical neck pain patients - Private practice	58 (28/30)	Lumbar SMT vs. Thoracic SMT - Remote region	Objective: Cervical pressure pain threshold and upper extremity grip strength - Yes	1	-
Karas et al.^32^	- USA and Germany - Private physiotherapy practice	- Adult mechanical neck pain patients (duration not specified) - Out-patient practice	69 (34/35)	Thoracic SMT (restriction) vs. Thoracic SMT (counter-restriction) - Same region	Subjective: Cervical pain intensity and disability - Yes	2	Daily: a series of home exercises—Restriction specific
Romero Del Rey et al.^30^	- Spain - Private physiotherapy practice	- Adult chronic mechanical neck pain patients - Private practice	186 (93/93)	Upper cervical SMTvs. Lower cervical and thoracic SMT - Remote region	Subjective: Cervical pain intensity - Yes	1	-
de Oliveira et al.^31^	- Brazil - Private physiotherapy practice	- Adult non-specific chronic low back pain patients - Private practice	148 (74/74)	Lumbar SMT vs. Thoracic SMT - Remote region	Subjective: lumbar pain intensity, disability, and global perceived change Objective: Cervical pressure pain threshold - Yes	10	-

### Methodological quality and risk of bias

This area of research was considered to be credible based on RoB and quality. As shown in Table 2, the studies could be considered high quality, as nearly all achieved “yes” on the four domains (7/10). Specifically, all reported a valid outcome and included a reproducible statistical description.

**Table 2:** Quality and risk of bias assessment of 10 studies included in a systematic review comparing the outcome of applying spinal manipulative therapy at a candidate site versus a non-candidate site.

References	Well described SMT technique - Type SMT used for the candidate site - Type SMT used for the non-candidate site	Description of how the candidate site was determined	Clinician qualified - Qualification - Years of experience	Outcome measurements reported to be reliable or reproducible	Transparent statistical approach to analysis	Number of correct quality items	Overall Risk of bias assessment
Haas et al.^23^	No - Unknown - Unknown	Palpation: endplay assessment	Yes - Two chiropractors - 20 and 2 years	Yes	Yes	3/4	Low
Cleland et al.^24^	Yes - Side-lying thrust with the painful side up - Non-specific supine thrust	Clinician selected: the painful side up, not specified further	Yes - 17 Physiotherapists - 9.1 mean years (SD = 5.9)	Yes	Yes	4/4	Low
Sutlive et al.^25^	Yes - Side-lying neutral-gap with the painful side up - Non-specific supine thrust	Clinician selected: the painful side up, not specified further	No - Unknown number of physiotherapists - Not reported	Yes	Yes	3/4	Low
Martinéz-Segura et al.^26^	Yes - Supine ipsilateral rotational thrust - Non-specific supine thrust	Palpation: pain localization and joint hypomobility	Yes - One physiotherapist - > 10 years (5 year with SMT)	Yes	Yes	4/4	Low
de Oliveira et al.^27^	Yes - Side-lying thrust - Non-specific supine thrust	Clinician selected: not specified further	Yes - One physiotherapist - 4.5 years	Yes	Yes	4/4	Moderate
Karas and Olson Hunt^28^	Yes - Supine thrust - Non-specific seated thrust	Palpation: joint hypomobility	Yes - Three physiotherapists - 13 mean years	Yes	Yes	4/4	High
Bautista-Aguirre et al.^29^	Yes - Supine thumb-move - Supine thrust	Participant: pain perception Palpation: joint hypomobility	Yes - One specialist manual therapist - Not reported	Yes	Yes	4/4	Moderate
Karas et al.^32^	Yes - Supine thrust - Supine thrust	Palpation: joint hypomobility	Yes - Eight physiotherapists - 18.1 mean years	Yes	Yes	4/4	Moderate
Romero Del Rey et al.^30^	Yes - Supine thrust - Multiple techniques	Test: Flexion-Rotation Test	Yes - One physiotherapist - > 9 years	Yes	Yes	4/4	Low
de Oliveira et al.^31^	Unclear - Side-lying thrust - Non-specific supine thrust	Clinician selected: not specified further	Yes - One physiotherapist - 11 years	Yes	Yes	3/4	Moderate

**Figure 2:**
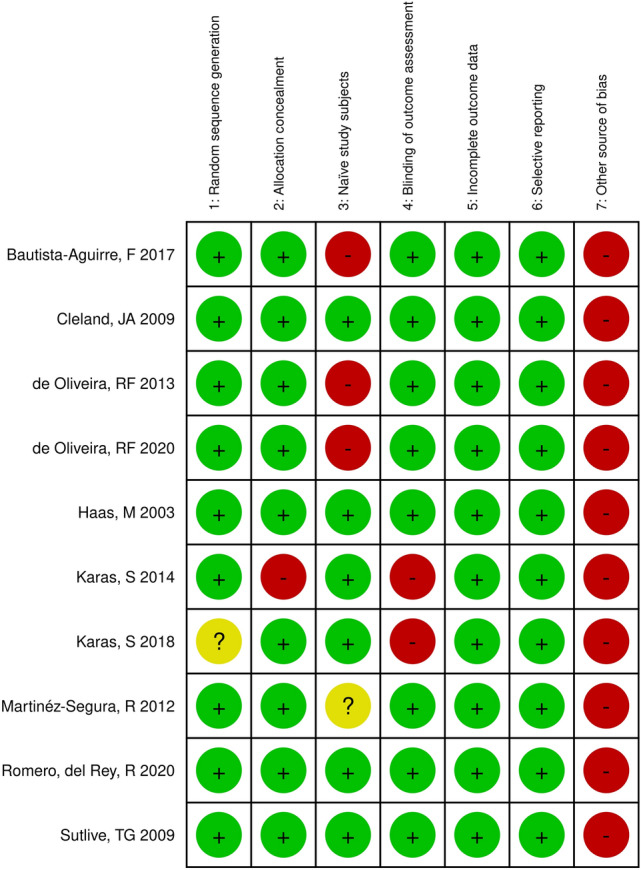
Summary of risk of bias for 10 studies in a systematic review comparing the outcome of applying spinal manipulative therapy at a candidate site versus a non-candidate site. The risk of bias was assessed using a modified version of the Cochrane Risk of Bias Tool for Randomized Controlled Trials. A ‘green + ’ indicates low risk of bias, a ‘red –’ indicates high risk of bias, and a ‘yellow ?’ indicates an unsure risk of bias.

**Figure 3:**
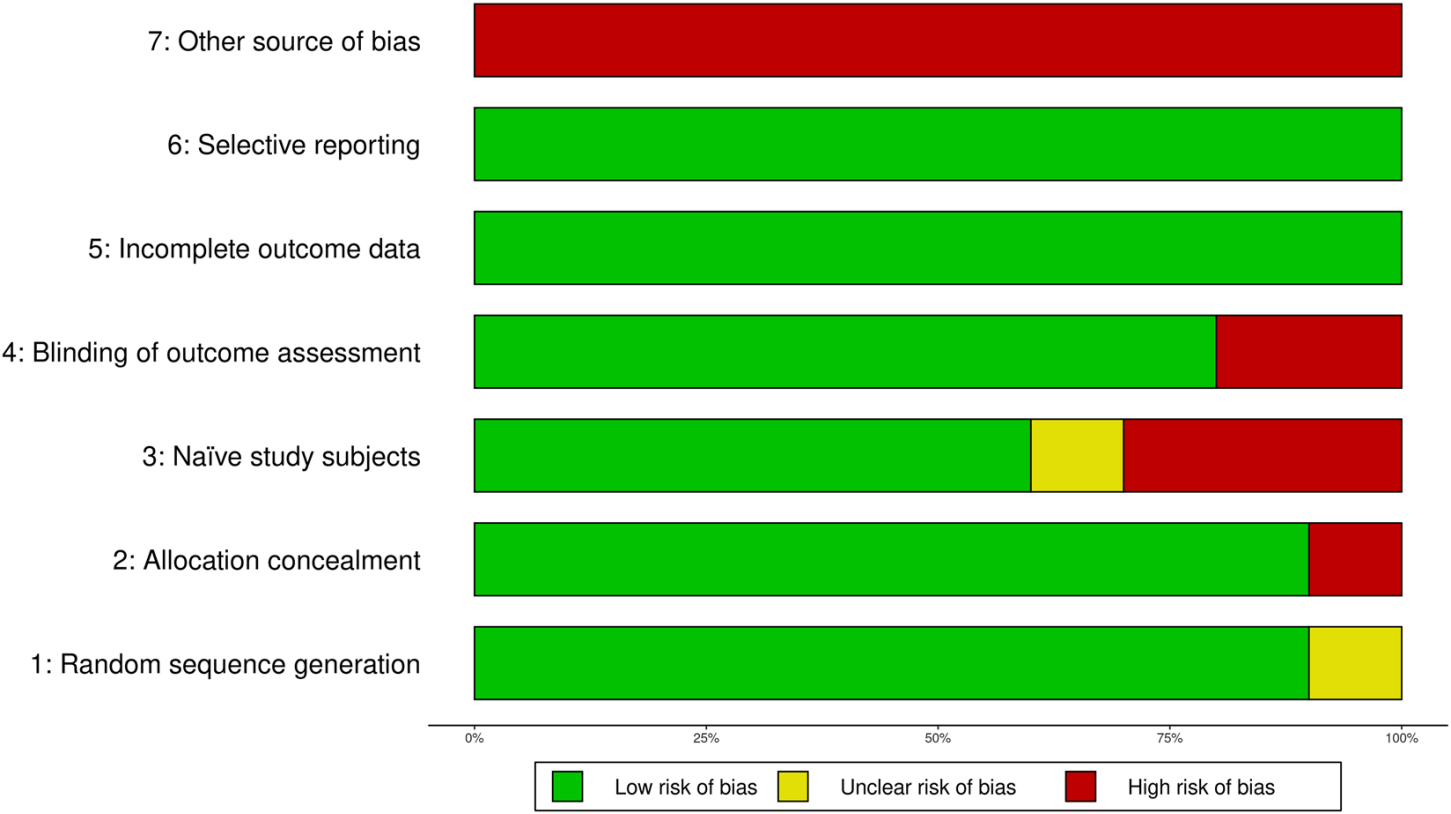
Risk of bias for each item across 10 studies included in a systematic review comparing the outcome of applying spinal manipulative therapy at a candidate site versus a non-candidate site. The risk of bias was assessed using a modified version of the Cochrane Risk of Bias Tool for Randomized Controlled Trials. ‘Green’ indicates low risk of bias, ‘red’ indicates high risk of bias, and ‘yellow’ indicates an unsure risk of bias.

Figure 2 shows that only one study was assessed as having high RoB^28^, four as moderate RoB^27,29,31,32^, and five as low RoB^23–26,30^. Items that commonly were deficient were “naïve study subjects” (to SMT) and “other sources of bias” (statistical analysis was performed blinded) (Fig. 3).

### SMT applied at the same vertebral level

Only one study of moderate RoB examined whether SMT outcomes differed when applied at the same vertebral level^32^. The candidate site was determined by a clinician using palpation for movement restriction, and the control SMT was applied at the same vertebra but in counter-direction. Outcomes were measured immediately following two SMT sessions and at two weeks follow-up. The study reported no between-group differences in neck pain or disability when comparing these two approaches.

### SMT applied in the same spinal region

Four studies applied SMT at a candidate site and SMT at a non-candidate site in the same spinal region. One study compared SMT at a candidate site to a random site^23^, and three to a non-specific or generalized regional SMT^24,25,28^ (i.e., the non-candidate SMT did not attempt to target a specific vertebral level).

In the first study^23^, assessed as low RoB, the clinician determined the candidate site by palpation assessing endplay and compared it to a non-candidate site determined as a matched random site in the cervical spine. Subjective neck pain, disability, and stiffness were measured immediately following one SMT session. No between-group differences were found.

Two studies assessed low back pain^24,25^, and the final study assessed neck pain^28^. The two low back pain studies were of low RoB, while the neck pain study was of high RoB. All outcomes were subjective and measured immediately after the first and only SMT session. However, one of the studies^24^ provided two sessions and repeated the measurements immediately following the second SMT session, at four weeks and 26 weeks. Only the neck pain study of high RoB^28^ reported a statistically significant but small between-group difference favoring the clinically relevant application, whereas the remaining two studies did not find any between-group differences.

### SMT applied in a remote spinal region

The remaining five studies compared SMT applied at a candidate site in the symptomatic area to SMT applied at a non-candidate site at a remote region^26,27,29–31^. Two investigated low back pain and compared SMT at the symptomatic lumbar spine versus SMT in the asymptomatic thoracic spine^27,31^. Two compared symptomatic cervical SMT to asymptomatic thoracic SMT for neck pain^26,29^, and one study examined upper cervical SMT to a series of SMTs at non-candidate sites: lower cervical, cervicothoracic, and mid-thoracic^30^.

The two low back pain studies^27,31^ were both of moderate RoB. The first study examined immediate changes following a single SMT session at the symptomatic lower back compared to the asymptomatic thoracic spine^27^. They found no between-group difference for patient-reported low back pain or PPT at the lumbar spine. The same author group reproduced this trial in 2020, now including ten SMT sessions instead and measured changes in subjective low back pain, disability, and global perceived change, as well as objective PPT at four, 12, and 26 weeks^31^. Again, there were no statistically significant differences, with all between-group differences close to 0 and with narrow confidence intervals.

Three studies assessed neck pain^26,29,30^. Two studies reported immediate changes. The first was of low RoB^26^ and compared cervical SMT at both the right and left side to thoracic SMT. As no between-group differences were reported between the left and right sides, we extracted results only from the right side (candidate site) compared to thoracic SMT (non-candidate site). This study found no between-group difference in neck pain. The second study of low RoB^30^ compared SMT at the candidate site (upper cervical vertebrae) to multiple SMTs at non-candidate sites and reported no between-group difference in neck pain intensity. The final study, which was of moderate RoB^29^, chose C7 as the candidate site (the clinician determined whether it was to be treated on the left or right side) and compared it to SMT at a non-candidate site (T3 level) for neck pain participants. No subjective outcomes were reported, only multiple PPTs across both upper limbs and bilateral grip strength immediately following one SMT session. We extracted only the initial PPT assessment (right wrist) and grip strength for the right hand. The between-group differences were not statistically significant.

### Summary of results

All results are reported in Table 3. We extracted a total of 33 between-group differences from ten studies. From these, nine studies^23–27,29–31^ (31 comparisons) reported no statistical between-group differences (low/moderate RoB, acceptable quality). Only one study^28^ (two comparisons) statistically favored SMT applied at the candidate site compared SMT at a non-candidate site for neck pain (mean difference of 1.2 out of 10 points (95% confidence interval = − 1.9 to − 0.5)) (high RoB, acceptable quality). Side effects were either not reported or were minimal and did not differ between groups receiving SMT at candidate and non-candidate sites.

**Table 3:** Results from 10 studies included in a systematic review comparing the outcome of applying spinal manipulative therapy at a candidate site versus a non-candidate site.

References	Number of participants reporting side effects Total (Clinically relevant SMT/control SMT)	Between group differences extracted/calculated	Assessed as a credible study (Yes/No) (i.e., low/moderate RoB and acceptable quality)	Re-tested whether the participants recognized that SMT was applied at the non-candidate site	Summary of results showing statistically significant clinical differences between the two treatment approaches
Haas et al.^23^	Not reported	Pain intensity [0 to 100] mean(SD): *Immediately* = *0.9 (3.5), Later same day* = *2.2 (3.5)* Subjective stiffness [0 to 100] mean (SD): I*mmediately* = *1.3 (3.4), Later same day* = *4 (3.6)*	Yes	Not reported	No between-group differences
Cleland et al.^24^	18 (9/9)	Pain intensity [0 to 10] mean [95%CI]: 1 week = *0.6 [*− *0.2, 1.4], 4 weeks* = *0.5 [*− *0.6, 1.5], 26 weeks* = *0.2 [*− *0.6, 1.0]* Disability[0 to 50] mean[95%CI]: *1 week* = *3.5 [*− *2.0, 9.0], 4 weeks* = *1.5 [*− *4.1, 7.1], 26 weeks* = − *0.9 [*− *5.5:3.8]*	Yes	Not reported	No between-group differences
Sutlive et al.^25^	Not reported	Pain intensity [0 to 1] effect size: *2 days* = *0.10* Disability [0 to 1] effect size: *2 days* = *0.23*	Yes	Not reported	No between-group differences
Martinéz-Segura et al.^26^	2 (1/1)	*Pain intensity [0 to 1] effect size:* *Immediately* = *0.06*	Yes	Not reported	No between-group differences
de Oliveira et al.^27^	0 (0/0)	Pain intensity [0 to 10] mean [95%CI]: *Immediately* = *0.5 [*− *0.1:1.1]* PPT lumbar [0:100] mean [95%CI]: *Immediately* = − *1.8 [*− *6.4:2.8]*	Yes	Not reported	No between-group differences
Karas and Olson Hunt^28^	Not reported	Pain intensity during cervical flexion [0 to 10] mean [95%CI]: *Immediately* = − *1.2 [*− *1.9:*− 0.5]* Cervical range of motion, flexion [0 to inf] mean [95%CI]: *Immediately* = *2.1 [*− *1.8:6.1]*	No	Not reported	A between-group difference was observed for cervical pain intensity immediately following treatment favoring the clinically relevant SMT
Bautista-Aguirre et al.^29^	Not reported	PPT wrist, right [0 to inf] mean [95%CI]: I*mmediately* = *0.0 [*− *1.4 to 1.8]* Grip strength, right [0 to inf] mean [95%CI]: I*mmediately* = *0.1 [*− *1.1 to 1.3]*	Yes	Not reported	No between-group differences
Karas et al.^32^	Not reported	Pain intensity [0 to 1] effect size: *2 days* = *0.25, 2 weeks* = *0.14* Disability [0 to 1] effect size: 2 days = *0.33, 2 weeks* = *0.18*	Yes	Not reported	No between-group differences
Romero Del Rey et al.^30^	Not reported	Pain intensity [0 to 1] effect size: 15 days = 0.00	Yes	Not reported	No between-group differences
de Oliveira et al.^31^	4 (0/4)	Pain intensity [0 to 10] mean [95%CI]: *4 weeks* = *0.0 [*− *0.9:0.9], 12 weeks* = − *0.1 [*− *1.0:0.8], 26 weeks* = − *0.1 [*− *1.0:0.8]* Disability [0 to 24] mean [95%CI]: *4 weeks* = *0.1 [*− *1.7:1.5], 12 weeks* = *0.1 [*− *1.6:1.7], 26 weeks* = − *0.9 [*− *2.5:0.7]* Global perceived change [− 5 to 5] mean [95%CI]: *4 weeks* = − *0.1 [*− *1.0:0.8], 12 weeks* = *0.3 [*− *0.7:1.2], 26 weeks* = *0.8 [*− *0.2:1.7]* PPT lumbar [0 to 2000] mean [95%CI]: *4 weeks* = *6 [*− *88:101]*	Yes	Not reported	No between-group differences

PPT, pressure pain detection threshold; 95%CI, 95% confidence intervals.

*reported as a statistically significant between-group difference.

## Discussion

### Statement of principal findings

This systematic review included ten randomized controlled clinical studies, of which nine were considered to have credible results. None of these nine studies detected any statistically significant differences in the 31 outcome measurements for the two treatment approaches. In other words, SMT given at a clinician-determined “correct” vertebral level did not have better outcomes than treatment given more haphazardly. These outcome measurements included pain, disability, and other objective outcomes. The only study to confirm the importance of treating the clinically relevant segment reported a small reduction in neck pain (1.2 points on an 11-point numerical rating scale)^28^. Although the magnitude of this effect is below the threshold for a minimally clinically important difference in this population^35^, the finding was statistically significant. However, that study was the only one assessed as having high RoB, which questions the validity of this result.

### Methodological considerations

#### Strengths and weaknesses of this review

Our review had several strengths: We independently selected the studies and data extraction protocols. We cannot exclude the possibility that other relevant publications have been missed. However, as the manual perusal of reference lists resulted in only one additional study, our search was likely near exhaustive. In addition, one RoB assessment criterion (item iii) was amended to reflect actual participant blinding. Although the modification of the RoB and the addition of the quality items is an approach that has not undergone careful external validation, the modification is uncomplicated and meaningful. As it is a methodological adjustment that fits the current study types, it is probably more a strength than a potential weakness. Also, a different approach is unlikely to have resulted in a different overall assessment of the credibility.

Many of the included studies did not provide estimates for their between-group mean differences. Therefore, instead of omitting the data, we calculated effect sizes from the mean within-group changes. However, this approach may have introduced errors as we had no means of confirming the underlying statistical assumptions for such calculations, particularly relevant for small samples, where the data could be skewed, heteroscedastic, or include outliers^12,13^. For that reason, we opted to make this approach only for the primary outcome (i.e., the patient-reported outcomes).

The systematic search was intentionally sensitive, as we expected a broad range of study methods. When considering the heterogeneity in both study design and outcome measurements, a meta-analysis was not feasible to conduct. However, it could also be argued that this heterogeneity is a strength of the review, as all the outcomes, except one, nevertheless follow the same pattern. The lack of pooling, not possible with such a small number of studies in each subgroup, also precluded any statistical modeling (e.g., exploring other factors) that may explain the lack of effects such as technique, thrust direction, speed, and how the candidate site was selected or patient characteristics, such as pain duration. Also, we expected multiple different outcomes to be reported, which is why we did not limit ourselves to any specific outcomes but extracted what was reported in the included studies.

#### Strengths and weaknesses of the included studies

Nine of the ten included studies were assessed as credible. Considering the RoB assessment, it is important to notice that blinding of participants and personnel is impossible in trials comparing SMT at two different regions^36^, so we removed this domain and considered instead whether the participants were naïve to SMT or not. Thus, instead of being blind to the type of treatment, the subjects should not have had a pre-determined idea of where and how SMT should be best applied. The issue is that only a few studies that we reviewed reported clearly to have taken this into account, which is probably a weakness of the studies that did not report (or consider) this version of participant blinding. However, we argue that the presence of this potential bias should have increased the likelihood that SMT applied at a candidate site being more effective. On the contrary, the studies generally did not find any between-group differences, and we consider that this further confirms our conclusion. Additionally, no studies reported whether the participants could infer if they received SMT at the candidate or non-candidate site.

A strength of the studies was the methodologically and reproducible trials, however, this is also a weakness as most studies investigated a single intervention (often a single session of SMT) in patients with chronic pain. Thus, the lack of difference between groups could perhaps be explained by i) the short duration of the intervention and ii) the clinical presentation.

### Clinical interpretation

To our knowledge, this is the first systematic review to explore the importance of the specificity of the application site of SMT in relation to clinical outcomes. As such, we are not able to compare these results against other studies. Our review advances evidence in this field and provides a more rigorous methodology to other narrative syntheses or evidence from individual studies on the subject.

The current systematic review failed to find any measurable difference in clinical outcome measurements based on whether the SMT was applied at a vertebral level based on clinical assessment (e.g., motion palpation) or not. This may run counter to the expectations and clinical experiences of those engaged in SMT. However, on reflection, this finding should not be surprising for several reasons.

#### The candidate site is a subjective concept

There are many lines of thinking regarding what tests to use to detect these presumed clinically relevant candidate sites to apply SMT^5^. Alas, there appear to be no studies that have succeeded in showing that such tests are reliable and reproducible. At the same time, it might be possible to locate a block vertebra using motion palpation^37^, and one chiropractor was able to recognize untreated patients by using this examination method, it was not possible to identify the treated patients^38^. Further, motion palpation cannot reliably distinguish between individuals from the general population with or without low back pain^39^. More recently, a systematic review recommended against the use of stand-alone tests for segmental motion assessment in patients with LBP^6^. Until demonstrated otherwise, reliable identification of a clinically relevant segment using manual assessment must be considered dubious.

Therefore, the detection method applied will depend on the profession, school of training, the fashion at the time of training, and own experience and preference. It is possible that, perhaps, clinically relevant candidate sites exist, but clinicians are unable to find them, which may explain the lack of difference in outcome between study groups. Therefore, the outcome in both groups may reflect not similarly promising results but similarly poor results. Thus, the results may simply capture the natural course of the condition in both groups at the time of assessment and indicate that the clinically relevant application site for SMT may, at present, be a nonsense concept. This is further supported by recent work concluding that the application site is not important for clinical outcomes despite attempting to target objectively determined clinically relevant sites, either in relation to stiffness or pain sensitivity^40^.

#### The manipulation is not specific

Another explanation relates not to the questionable validity of test procedures but in attempting to perform a *specific* SMT procedure. It has been shown that SMT has a wider effect on multiple vertebral joints, both in proximity and further away from the application site. Studies in which accelerometers or microphones have been used to record the location of the “crack”-sound associated with SMT have found that it does not necessarily stem from the SMT application site^41–43^. It is not obvious how to interpret such findings, but they certainly do not suggest that the mechanical effects of SMT are restricted to the application site.

#### A neuromuscular or biomechanical mechanism might explain the positive results of SMT

The positive changes observed after SMT may be unrelated to treatment specificity but an effect of a generalized (systemic) effect or biomechanical interactions, such as functional changes in a “biomechanical chain” and spinal regional interdependence^44^. This could explain why thoracic SMT seems to reduce cervical pain in clinical adult populations^45–47^. Examples of other potential biomechanical effects are increased disc diffusion and decreased posterior-anterior stiffness^48^. Other systemic effects could include changes in the functioning of descending anti-nociceptive system^49^, a widespread effect on muscle spindle response^50^, and central mechanisms of pain modulation^51^. These examples are not an exhaustive list of potential mechanisms, as this topic is outside the scope of this systematic review. Possibly, the benefits of SMT might come from mechanisms that have not yet been investigated thoroughly^52^ or complex interactions that cannot currently be understood.

#### Contextual contributions might explain the positive results of SMT

It is possible that at least some positive effects of SMT may be due to non-specific mechanisms such as contextual contributions (e.g., patient expectations and a response to the therapeutic alliance)^53,54^. These systemic and non-specific factors could contribute to an increased improvement following SMT. The same has been observed in acupuncture^55^ and exercise^56^, and it is a general finding across multiple interventions^57^. The same argument can be made for SMT in general, as it is non-superior to non-thrust mobilization or even sham SMT^58^. Thus, the application site (e.g., spinal level) and application type (high velocity, low amplitude or mobilization) would not be central to successful manual therapy. The results of this systematic review support this statement.

#### A more nuanced theory

Thus, while SMT appears to be an efficient intervention in some with spinal pain conditions^58,59^, the choice of the application site does not appear to modify this effect, and a more nuanced theory of treatment mechanism must account for this observation. Finally, these findings apply to all manual therapist professions as the efficacy of SMT does not appear to be therapist-dependent^58^.

### Unanswered questions and future research

#### Future research

We acknowledge that further research is required to determine the underlying mechanisms of SMT. However, as clinicians cannot quantify or reliably locate spinal dysfunctions suitable for SMT application, clinicians must accept that the choice of SMT application site is based on an entirely subjective decision process. Therefore, there appears to be limited value in conducting further trials striving to optimize SMT by comparing specific applications as an intervention for spinal pain, at least until our knowledge of SMT mechanisms has improved.

#### Educational institutions

This review does not contradict the teaching and clinical use of SMT. However, it suggests that the best available evidence does not emphasize technical concepts of specificity related to improving clinical outcomes. We recommend that curricula should include how “non-specific SMT” can be used advantageously.

## Conclusions

The current evidence does not support that SMT applied at a supposedly “clinically relevant” candidate site is superior to SMT applied at a supposedly “not clinically relevant” site for individuals with spinal pain. Whether this is true for objective outcomes is unknown. A more nuanced model related to the concept of specificity in spinal manipulation needs to be established and systematically tested for validity.

## Supplementary Information

Supplementary Information 1.

Supplementary Information 2.

Supplementary Information 3.

## Data Availability

All data are available in Supplementary information 2.
