# Supplementary material for: The importance of selecting the correct site to apply spinal manipulation when treating spinal pain: Myth or reality? A systematic review
Source: Sci Rep. 2021 Dec 3;11:23415. doi: 10.1038/s41598-021-02882-z (PMC8642385; doi:10.1038/s41598-021-02882-z)
Supplement: Supplementary file 1 — Supplementary Information 1. [file 41598_2021_2882_MOESM1_ESM.pdf]

**PubMed**
((("Musculoskeletal Pain"[Mesh] OR "Musculoskeletal Pain" OR "neck pain"[MeSH Terms] OR "Neck Pain" OR "Back Pain"[Mesh] OR "Low Back Pain" OR "Back Pain" OR "Thoracic pain" OR "spinal pain") AND ("Musculoskeletal Manipulations"[Mesh] OR manipu* OR "spinal adjust*" OR chiro* OR osteopath*)) AND (Segment OR level OR region OR specific OR site OR vertebra* OR clinic* OR direct*)) AND (gener* OR random OR nonspecific OR non-specific OR multiple OR prescript* OR non-region OR distant)

**Embase**

1. musculoskeletal pain.mp. or 'musculoskeletal pain'.ti,ab,kw. or neck pain.mp. or 'neck pain'.ti,ab,kw. or low back pain.mp. or 'low back pain'.ti,ab,kw. or thorax pain.mp. or 'thoracic pain'.ti,ab,kw. or backache.mp. or spinal pain.mp. or 'back pain'.ti,ab,kw. or 'spinal pain'.ti,ab,kw. 269643
2. manipulative medicine.mp. or 'manipulative medicine'.ti,ab,kw. or manipu*.ti,ab,kw. or 'spinal adjust*'.ti,ab,kw. or chiro*.ti,ab,kw. or osteopath*.ti,ab,kw.273730
3. Segment.ti,kw,ab. or level.ti,ab,kw. or specific.ti,ab,kw. or site.ti,ab,kw. or vertebra*.mp. or vertebra*.ti,ab,kw. or clinic*.ti,ab,kw. or direct*.ti,ab,kw. 13782273
4. (gener* or random or nonspecific or non-specific or multiple or prescript* or non-region or distant).ti,ab,kw.

Combine with AND

**CINAHL**
((MH "Muscle Pain") OR (MH "Low Back Pain") OR (MH “Back Pain”) OR (MH “Neck Pain”) OR “Low Back Pain“ OR “Back Pain” OR “Thoracic pain” OR “spinal pain” OR Neck pain ) AND ( (MH "Manual Therapy") OR manipu* OR "spinal adjust*" OR chiro* OR osteopath* ) AND ( (MH "Lumbar Vertebrae") OR (MH "Thoracic Vertebrae") OR (MH "Cervical Vertebrae") Segment OR level OR region OR specific OR site OR vertebra* OR clinic* OR direct* ) AND ( gener* OR random OR nonspecific OR non-specific OR multiple OR prescript* OR non- region OR distant )

**Index to chiropractic literature**

1. - Subject:Musculoskeletal Pain OR All Fields:“Musculoskeletal Pain”, Peer Review only OR Subject:\"Neck Pain\", Peer Review only OR Subject:\\\"Low Back Pain\\\" OR Subject:\"Back Pain\", Peer Review only OR All Fields:Neck Pain OR All Fields:Back Pain OR All Fields:Low Back Pain OR All Fields:Thoracic pain OR All Fields:spinal pain
2. - Subject:\\\"Musculoskeletal Manipulations\\\" OR Subject:\\\"Manipulation, Chiropractic\\\" OR All Fields:spinal adjust* OR All Fields:chiro* OR All Fields:osteopath*
3. - All Fields:Segment OR All Fields:level OR All Fields:region OR All Fields:specific OR All Fields:site OR All Fields:vertebra* OR All Fields:clinic* OR All Fields:direct* OR Subject:\\\"Cervical Vertebrae\\\" OR Subject:\\\"Thoracic Vertebrae\\\" OR Subject:\\\"Lumbar Vertebrae\\\"
4. - All Fields:gener* OR All Fields:nonspecific OR All Fields:non-specific OR All Fields:distant OR All Fields:distant OR All Fields:random OR All Fields:multiple OR All Fields:prescript* OR All Fields:non-region OR All Fields:distant

Combine with AND
